# The outcome of COVID‐19 patients in the intensive care unit in Sudan: A cross‐sectional study

**DOI:** 10.1002/hsr2.1161

**Published:** 2023-03-22

**Authors:** Mohammed A. O. Ali, Noon A. Abdalrahman, Elaf A. I. Shanab, Mozan M. A. Mohammed, Malaz M. Ibrahim, Ihab B. Abdalrahman

**Affiliations:** ^1^ Faculty of Medicine University of Khartoum Khartoum Sudan; ^2^ Faculty of Medicine Ahfad University for Women Omdurman Sudan

**Keywords:** COVID‐19, ICU, intubation, mortality, Sudan

## Abstract

**Background and Aim:**

A major outbreak of coronavirus spread all over the world and gave rise to high mortality rate and high admission rate to intensive care unit (ICU). This cohort study aims to assess the outcome of COVID‐19 patients in ICU and to investigate the factors associated with mortality.

**Method:**

This is a multicentered retrospective cohort study that was conducted among confirmed cases of COVID‐19 patients, who were admitted to ICU in Sudan during March 2021. The data collection was done manually from the medical records of patients. Mortality rate and association and prediction of factors associated with mortality were obtained using Statistical Package for the Social Sciences software (SPSS) version 22.

**Results:**

The mortality rate among patients in this study was 70%. Using the chi‐square test we found that age, needing intubation, developing Systemic inflammatory response syndrome, neurological complications, hematological complications, and cardiac complications have a significant association with the outcome.

**Conclusion:**

Majority of COVID‐19 patients who were admitted to the ICU died. 55.8% of patients developed at least one complication during their stay in ICU. The age, the need for intubation, and developing of systematic inflammatory response syndrome (SIRS) are the factors that predict the mortality.

## 1 INTRODUCTION

The coronavirus (COVID‐19) outbreak began in Wuhan, China, in late December 2019, and then spread to nearly every country in the world.^1^ In March 2020, it was declared a pandemic by the WHO.^2^ With more than 200 million reported cases and more than 4 million deaths, it has become one of the most critical health emergencies in the world.^3^

Despite the slow arrival of this disease to Africa, the impact of COVID‐19 on these vulnerable populations was very burdensome, causing disruptions to the health system in addition to significant costs to the economy.^4^, ^5^

Severe acute respiratory syndrome coronavirus 2 (SARS‐CoV‐2) is the causative agent of this viral infection, which primarily affects the respiratory system in addition to multiorgan involvement. The clinical manifestations of the disease range from asymptomatic infection to severe illness and even multiorgan failure and death.^6^

Most studies have observed that poor outcomes are related to old age and the presence of comorbidities such as diabetes, hypertension, COPD, and other chronic diseases.^7^, ^8^ Populations having these factors are vulnerable to high hospitalization and ICU admission rates.^9^ Those admitted to the ICU have an even higher rate of morbidity and mortality with the development of severe complications such as sepsis, acute respiratory distress syndrome, pulmonary embolism, multiple organ failure, neurological complications, and others, apart from the need for mechanical ventilation.^9^, ^10^

Comprehensive data of COVID‐19 patients who are admitted to the ICU are needed to help healthcare providers to adopt appropriate management and interventions, to aim health authorities at national and international levels to develop various strategies that help to reduce mortality rates and combat this pandemic to save lives with limited resources. But unfortunately, in Sudan, COVID‐19 data are limited, with no computerized medical records of patients making the issue of research difficult.

This cohort study aims to assess the outcome of COVID‐19 patients in ICU and to investigate the factors associated with mortality.

## 2 METHODOLOGY

### 2.1 Study design and study population

This is a multicentered retrospective cohort study that was conducted among confirmed cases of COVID‐19 patients, who were admitted to ICU in Sudan during march 2021.

The data were collected from medical records documented in ICU of both private and general hospitals in Sudan. Eight hospitals were included in this study, of which 6 hospitals were private. All patients aged 18 years and above, diagnosed with COVID‐19 using polymerase chain reaction (PCR), and admitted to the ICU during the study period were included in this study. It is worth mentioning that hospitalized cases of COVID‐19 who were not admitted to the ICU were not included in this study. Unfortunately, during the study period, the COVID‐19 vaccine was just introduced in Sudan so almost all of the patients were not vaccinated.

### 2.2 Data collection

The collection of data was done manually from the medical records of patients using data sheets composed of three parts. The first part is about the patients’ characteristics such as age, gender, comorbidities, duration of stay in ICU, the need for intubation, The second part is about the complication, and the third part is about the outcome.

### 2.3 Data analysis

The Statistical Package for the Social Sciences software (SPSS) version 22 was used to analyze this data. Categorical data were reported as frequencies and percentages, while continuous data were not normally distributed and were reported as the median and interquartile range (IQR). Figures and tables were used to display the data. To find the association between the categorical dependent and the independent variables, we used Pearson's chi‐square test (two‐sided asymptotic value obtained). Furthermore, we measured the prediction of the independent variables using binary logistic regression. Any *p*‐value equals or less than 0.05 was considered to be statically significant for all analyses.

### 2.4 Ethical considerations

Ethical approval was obtained from the ministry of health before starting the data collection. Participants’ consent was obtained by contacting their families through their registered phone numbers.

## 3 RESULTS

### 3.1 Demographics and clinical characteristics of patient

A total of 217 patients who met the selection criteria during the study period were included in this study. Most of those patients were male (64.5%), with a median age of 63 years (IQR: 57–66). 50.7% of them are above 65 years. Surprisingly, 77.4% of those patients are having at least one comorbidity, of which hypertension (55.3%) and diabetes (46.5%) are the most common chronic diseases existed among them. Moreover, 58.5% of those patients need to stay more than 7 days in the ICU with a median duration of 11 days (IQR: 9–14). Despite this long period, only 36.9% of those patients required intubation (Table 1).

**Table 1:** Association between patient's factors and the outcome.

	Outcome				
Variables	Discharge	Death	Total *n* (%)	*χ*²	*p*‐value
Gender					
Male	38	102	140 (64.5%)	1.486	0.278
Female	27	50	77 (35.5%)		
Age					
Below 65 years	44	66	110 (50.7%)	10.731	**0.001**
Above 65 years	21	86	107 (49.3%)		
HTN					
Yes	33	87	120 (55.3%)	0.770	0.456
No	32	65	97 (44.7%)		
Diabetes					
Yes	26	75	101 (46.5%)	1.597	0.236
No	39	77	116 (53.4%)		
Heart disease					
Yes	6	27	33 (15.2%)	2.571	0.148
No	59	125	184 (84.8%)		
Malignancy					
Yes	2	7	9 (4.1%)	0.268	0.728
No	63	145	208 (95.9%)		
CKD					
Yes	3	16	19 (8.8%)	1.991	0.196
No	62	136	198 (91.2%)		
Asthma					
Yes	3	10	13 (6%)	0.312	0.759
No	62	142	204 (94%)		
COPD					
Yes	1	4	5 (2.3%)	0.242	1.000
No	64	148	212 (97.3%)		
Lung fibrosis					
Yes	0	5	5 (2.3%)	2.189	0.325
No	6	147	212 (97.7%)		
Liver disease					
Yes	2	5	7 (3.2%)	0.007	1.000
No	63	147	210 (96.8%)		
Free medical background					
Yes	18	31	49 (22.6%)	1.387	0.228
No	47	121	168 (77.4%)		
Stay in intensive care unit (ICU)					
More than 7 days	34	93	127 (58.5%)	1.478	0.233
Less than 7 days	31	59	90 (41.5%)		
Need intubation					
Yes	1	79	80 (36.9%)	49.759	**0.001**
No	64	73	137 (63.1%)		

Unfortunately, 55.8% of patients developed at least one complication during their stay in ICU, with systemic inflammatory response syndrome (SIRS) (48.4%) being the most common complication that happened among them (Table 2).

**Table 2:** Association between complications that occur during intensive care unit (ICU) admission and the final outcome.

Variables	Outcome				
	Discharge	Death	Total *n* (%)	*χ*²	*p*‐value
SIRS^a^					
Yes	1	104	105 (48.4%)	81.552	**0.001**
No	64	4	112 (51.6%)		
Neurological^b^					
Yes	1	17	18 (8.3%)	7.888	**0.004**
No	64	135	199 (91.7%)		
Hematological^c^					
Yes	0	29	29 (13.4%)	14.314	**0.001**
No	65	123	188 (86.6%)		
Cardiac^d^					
Yes	0	17	17 (7.8%)	7.888	**0.004**
No	65	135	200 (92.2%)		
Bedsore					
Yes	0	7	7 (3.2%)	3.093	0.106
No	65	145	210 (96.8%)		

^a^ *Note*: Bold values are statistically significant at *p* values.

^a^ Systemic inflammatory response syndrome.

^b^ Stroke, delirium.

^c^ Anemia, Bleeding.

^d^ Arrythmia, myocardia infraction.

### 3.2 Outcome and association

The mortality rate among patients in this study was 70%. only 30% of patients were discharged home well. Plotting factors in Tables 1 and 2 against the outcome, we found that age, needing intubation, developing SIRS, neurological complications, hematological complications, and cardiac complications have a significant association with the outcome using the chi‐square test (Tables 1 and 2).

### 3.3 Multivariate analysis

Intending to find which factor could predict mortality, we entered all the associated factors in Tables 1 and 2 into the binary Logistic Regression analysis. Patients who devolved Systematic Inflammatory Response Syndrome (SIRS) were 81‐fold (95% confidence interval [CI]: 10.1–648.6) more likely to die than those who did not develop this syndrome. Additionally, the need for intubation carries a 46‐fold (95% CI: 5.4–400.1) risk of death in those patients who need intubation. As could be expected, being older than 65 years old gives a 3‐fold (95% CI: 1.2–8.7) risk of death than being younger (Table 3).

**Table 3:** Binary logistic regression analysis.

Variables	*p*‐value	Odd ratio	Confidence interval for odd ratio
Age	**0.013**	**3.357**	1.288–8.754
Systematic inflammatory response syndrome	**0.001**	**81.231**	10.172–648.668
Need Intubation	**0.001**	**46.693**	5.449–400.145
Neurological	0.068	0.997	0.850–99.638
Hematological	0.997	236484607.5	0.000
Cardiac	0.998	192225521.7	0.000
Constant	0.001	0.199	

*Note*: Bold values are statistically significant at *p* values.

## 4 DISCUSSION

### 4.1 The pattern of admission and mortality

This multi‐center retrospective cohort study was conducted among 217 confirmed cases of COVID‐19 who were admitted to ICU in Khartoum state. The infection of COVID‐19 was confirmed by the polymerase chain reaction testing (PCR) from the nasopharyngeal sample. The median age of patients was 63 (IQR: 57–66) years, indicating that elderly patients were admitted more to the ICU. Yet, those who aged more than 65 years were 49.3%, and those who were below 65 were 50.7%. Similarly, other studies showed median ages ranged between 55 and 74 years.^11^, ^12^, ^13^, ^14^, ^15^

Male participants in this study constituted 64.5% which is similar to the studies from Italy, Europe, Belgium, Sudan, and Mexico where more than 60% of the participants were males.^11^, ^12^, ^13^, ^16^, ^17^ This might give rise to a hypothesis that males do get infected more with COVID‐19 with a severe disease that renders them critically ill in the ICU, it is also consistent with the meta‐analysis that showed male sex as a risk factor for death and ICU admission due to COVID‐19 infection.^18^ Yet, there is no statistical significance was found between the male gender and mortality in this study.

The median ICU admission duration was 11 days (IQR: 9–14), which is similar to other reviewed studies where median ICU stay ranged between 6 and 12 days regardless of the mortality rate.

The mortality rate in this cohort was 70% which is higher than percentages recorded in the studies from Lombardy; Italy (48.3%), Europe (24.3%), Sudan (50%), the USA (39.5%), and Wuhan; China (61.5%) and slightly lower than mortality of Mexico (72.7%) and Belgium (72.0%).^9^, ^11^, ^12^, ^13^, ^15^, ^16^, ^17^

### 4.2 Impact of mechanical ventilation

Among all participants, 36.8% required invasive mechanical ventilation, this percentage is much lower than those documented in studies from Europe (49.3%),^12^ Belgium (63%),^13^ Wuhan; China (69%),^15^ Mexico (43%),^17^ and significantly higher than USA (19%).^9^ the significant observation is that 98.7% of those patients who require invasive mechanical ventilation were deceased, which would appear to indicate a strong association between invasive mechanical ventilation and mortality. The latter high percentage might be reflected by the complications of intubation in critically ill patients regardless of the illness. In Canada, a cohort study showed an increased rate of ICU complications and mortality in intubated patients in comparison with nonintubated patients.^19^ In Sudan, the rate of complication during ICU admission was found in several studies to be increased due to complications like infections and complications related to intubation.^20^, ^21^ Contrary to expectations, the Canadian cohort revealed no difference in outcome between intubated by experienced medical personnel versus those intubated by non‐experienced personnel.^19^

### 4.3 The pattern of complications

The most common complication reported after ICU admission in this cohort was SIRS that 48% of patients developed. This high percentage may be attributed to many factors including the immunological response to the COVID‐19 virus^22^ and ICU‐related infections.^23^ ICU admission itself, regardless of the illness, was found to be associated with the development of sepsis in many studies.^23^ Ventilation‐associated pneumonia reported in ICU critically ill patients is highly associated with the development of SIRS.^24^ Hence, the development of this syndrome could be multifactorial.

In this cohort, only 8% of patients developed neurological complications, this percentage is much lower when compared to the study conducted in the United Kingdom where 62% of participants developed neurological events both at presentation and during the hospital stay.^25^

Moreover, a global cohort study of the incidence of neurological complications among COVID‐19 patients revealed a prevalence of about 80% among them.^26^ Hematological complications occurred in 13.4% of the patients, while 7.8% developed cardiac complications. These percentages are similar to other studies from the United States and Europe.^9^, ^12^ The later complications are considered low and even significantly lower when compared with those who developed SIRS. The development of hematological complications can be attributed to the effect of the COVID‐19 virus on the hemostasis and coagulation profile as also shown in other studies.^27^, ^28^

Bedsores were reported in 3.2% of the patients during ICU stay, with the previously mentioned median stay duration of 11 days will question the proper nursing of patients in ICU. Moreover, bed sores themselves can act as a source of infection, which might contribute to the development of SIRS.^29^

### 4.4 Associations and predictions

Using the chi‐square test, age and the need for intubation were significantly associated with mortality, along with complications such as the development of SIRS, neurological, hematological, and cardiac complications after admission to ICU.

Furthermore, on binary logistic regression analysis, age (odds ratio [OR]: 3.357), need for intubation (OR: 46.693), and the development of SIRS (OR: 81.231) predicted the mortality of COVID‐19 patients. The finding of older age as a predictor for mortality is consistent with reviewed studies from Sudan (OR: 1.05) and Belgium (Odds OR: 1.07).^13^, ^16^

The development of SIRS is a predictor of mortality among COVID‐19 patients in this study, but this was not mentioned in other reviewed studies. Nevertheless, an increase or decrease in white blood cell count, which is a component of SIRS, was a predictor for mortality in studies from eastern Sudan and Mexico.^16^, ^17^

The usage of mechanical ventilation predicted higher mortality in this cohort. Although this intervention aims to save lives, it was found to be associated with mortality in other countries, the systematic review and meta‐analysis of the factors associated with mortality in COVID‐19 patients identified the need for invasive mechanical ventilation as highly associated with mortality (OR: 2.53, 95% CI: 1.90–3.37).^30^ The latter observation will highlight the need to delay the decision to intubate patients of COVID‐19, and saving this intervention for last resort.

Against all expectations, this cohort showed a lack of association between the presence of comorbidities and mortality, a result that is similar to another study from Sudan^16^ but also different from the rest of reviewed studies from Lombardy; Italy^11^ and Wuhan; China^15^ which showed having one or more comorbidity is highly associated with death. This contradiction is substantial and would suggest that comorbidities in COVID‐19 patients may have a low effect on mortality from COVID‐19. Based on such an observation, a subsequent alteration of practice or interventions in COVID‐19 patients may ensue. However, this finding needs to be interpreted with caution and proposed as a vital issue for further research.

### 4.5 Study limitations

As we mentioned, in Sudan, COVID‐19 data are limited, with no computerized medical records of patients making the issue of research difficult. Some medical records of patients were missed, with the fact that the access to all hospitals with ICU was difficult due to the hospital policy which prevent us to obtain the patients’ records. It is very important to mention that all patients included in this study are not vaccinated, so further assessments on the effect of vaccine on mortality of COVID‐19 patients in intensive care unit should be evaluated and compared to this study.

## 5 CONCLUSION

This study showed that most of COVID‐19 patients who were admitted to the ICU died. 55.8% of patients developed at least one complication during their stay in ICU. Using the chi‐square test we found that age, needing intubation, developing SIRS, neurological complications, hematological complications, and cardiac complications have a significant association with the outcome. Only age, the need for intubation, and developing SIRS predict the mortality.

## AUTHOR CONTRIBUTIONS

**Mohammed A. O. Ali**: Conceptualization; data curation; formal analysis; investigation; methodology; resources; validation; visualization; writing—original draft; writing—review and editing. **Noon A. Abdalrahman**: Writing—original draft. **Elaf A. I. Shanab**: Data curation; writing—original draft; writing—review and editing. **Mozan M. A. Mohammed**: Data curation. **Malaz M. Ibrahim**: Writing—original draft. **Ihab B. Abdalrahman**: Supervision.

## CONFLICT OF INTEREST STATEMENT

The authors declare no conflict of interest.

## TRANSPARENCY STATEMENT

The lead author Mohammed A. O. Ali affirms that this manuscript is an honest, accurate, and transparent account of the study being reported; that no important aspects of the study have been omitted; and that any discrepancies from the study as planned (and, if relevant, registered) have been explained.

## Data Availability

The data that support the findings of this study are available on request from the corresponding author. The data are not publicly available due to privacy or ethical restrictions.

## References

[1] WHO . *Archived: WHO Timeline ‐ COVID‐19 [Internet]*; 2021. https://www.who.int/news/item/27-04-2020-who-timeline---covid-19

[2] WHO . *WHO Director‐General's Opening Remarks at the Media Briefing on COVID‐19 ‐ 11 March 2020 [Internet]*; 2021. https://www.who.int/director-general/speeches/detail/who-director-general-s-opening-remarks-at-the-media-briefing-on-covid-19---11-march-2020

[3] WHO . *WHO Coronavirus (COVID‐19) Dashboard [Internet]*; 2021. https://covid19.who.int/

[4] Inzaule SC , Ondoa P , Loembe MM , Tebeje YK , Ouma AEO , Nkengasong JN . Covid‐19 and indirect health implications in Africa: impact, mitigation measures, and lessons learned for improved disease control. PLoS Med. 2021;18(6):e1003666. 10.1371/journal.pmed.1003666 34161318 PMC8266084

[5] Ataguba JE . Covid‐19 pandemic, a war to be won: understanding its economic implications for Africa. Appl Health Econ Health Policy. 2020;18(3):325‐328. 10.1007/s40258-020-00580-x 32249362 PMC7130452

[6] Samudrala PK , Kumar P , Choudhary K , et al. Virology, pathogenesis, diagnosis and in‐line treatment of covid‐19. Eur J Pharmacol. 2020;883:173375. 10.1016/j.ejphar.2020.173375 32682788 PMC7366121

[7] Chen Y , Klein SL , Garibaldi BT , et al. Aging in COVID‐19: vulnerability, immunity and intervention. Ageing Res Rev. 2021;65:101205. 10.1016/j.arr.2020.101205 33137510 PMC7604159

[8] Ejaz H , Alsrhani A , Zafar A , et al. Covid‐19 and comorbidities: deleterious impact on infected patients. J Infect Public Health. 2020;13(12):1833‐1839. 10.1016/j.jiph.2020.07.014 32788073 PMC7402107

[9] Kim L , Garg S , O′Halloran A , et al. Risk factors for intensive care unit admission and in‐hospital mortality among hospitalized adults identified through the US coronavirus disease 2019 (covid‐19)‐associated hospitalization surveillance network (COVID‐net). Clin Infect Dis. 2020;72(9):e206‐e214. 10.1093/cid/ciaa1012 PMC7454425 32674114

[10] Contou D , Cally R , Sarfati F , Desaint P , Fraissé M , Plantefève G . Causes and timing of death in critically ill covid‐19 patients. Crit Care. 2021;25(1):79. 10.1186/s13054-021-03492-x 33622349 PMC7901794

[11] Grasselli G , Greco M , Zanella A , et al. Risk factors associated with mortality among patients with COVID‐19 in intensive care units in lombardy, Italy. JAMA Int Med. 2020;180(10):1345‐1355. 10.1001/jamainternmed.2020.3539 PMC7364371 32667669

[12] Wendel Garcia PD , Fumeaux T , Guerci P , et al. Prognostic factors associated with mortality risk and disease progression in 639 critically ill patients with covid‐19 in Europe: initial report of the international RISC‐19‐ICU prospective observational cohort. EClinicalMedicine. 2020;25:100449. 10.1016/j.eclinm.2020.100449 32838231 PMC7338015

[13] van Halem K , et al. Correction to: risk factors for mortality in hospitalized patients with COVID‐19 at the start of the pandemic in Belgium: a retrospective cohort study. BMC Infect Dis. 2020;20(1):897. 10.1186/s12879-020-05690-4 33246431 PMC7691970

[14] Asch DA , Sheils NE , Islam MN , et al. Variation in US hospital mortality rates for patients admitted with covid‐19 during the first 6 months of the pandemic. JAMA Int Med. 2021;181(4):471. 10.1001/jamainternmed.2020.8193 PMC7756246 33351068

[15] Wang Y , Lu X , Li Y , et al. Clinical course and outcomes of 344 intensive care patients with covid‐19. Am J Respir Crit Care Med. 2020;201(11):1430‐1434. 10.1164/rccm.202003-0736le 32267160 PMC7258632

[16] Omar S , Musa I , Salah S , Elnur M , Al‐Wutayd O , Adam I . High mortality rate in adult COVID‐19 inpatients in eastern Sudan: a retrospective study. J Multidiscip Healthc. 2020;13:1887‐1893. 10.2147/jmdh.s283900 33324068 PMC7733440

[17] Abbattista M , Ciavarella A , Capecchi M , et al. Risk factors for mortality in hospitalized patients with COVID‐19: a study in Milan, Italy. Infect Dis. 2020;53(3):226‐229. 10.1080/23744235.2020.1859131 33307901

[18] Peckham H , de Gruijter NM , Raine C , et al. Male sex identified by global covid‐19 meta‐analysis as a risk factor for death and itu admission. Nat Commun. 2020;11(1):6317. 10.1038/s41467-020-19741-6 33298944 PMC7726563

[19] Griesdale DEG , Bosma TL , Kurth T , Isac G , Chittock DR . Complications of endotracheal intubation in the critically ill. Intensive Care Med. 2008;34(10):1835‐1842. 10.1007/s00134-008-1205-6 18604519

[20] Osman T . Prevalence of intensive care unit‐acquired infections in different hospitals in Khartoum state‐Sudan. Am J PharmTech Res. 2019;9(5):176‐186. 10.46624/ajptr.2019.v9.i5.015

[21] Ahmed AMA , Elkaki MME , Albadri AKE , Ali AH . Prevalence of hospital acquired pneumonia in intensive care units in Khartoum state (from April 2019 to September 2019). J Clin Biomed Res. 2021;3 (2):1‐4. 10.47363/jcbr/2021(3)130

[22] Polidoro RB , Hagan RS , de Santis Santiago R , Schmidt NW . Overview: systemic inflammatory response derived from lung injury caused by SARS‐COV‐2 infection explains severe outcomes in covid‐19. Front Immunol. 2020;11:11. 10.3389/fimmu.2020.01626 32714336 PMC7344249

[23] Sakr Y , Jaschinski U , Wittebole X , et al. Sepsis in intensive care unit patients: worldwide data from the intensive care over nations audit. Open Forum Infect Dis. 2018;5(12):ofy313. 10.1093/ofid/ofy313 30555852 PMC6289022

[24] Bonten MJM , Froon AHM , Gaillard CA , et al. The systemic inflammatory response in the development of ventilator‐associated pneumonia. Am J Respir Crit Care Med. 1997;156(4):1105‐1113. 10.1164/ajrccm.156.4.9610002 9351609

[25] Varatharaj A , Thomas N , Ellul M , et al. UK‐wide surveillance of neurological and neuropsychiatric complications of COVID‐19: the first 153 patients. SSRN Electron J. 2020. Preprint. 10.2139/ssrn.3601761 PMC7316461 32593341

[26] Chou SH‐Y , Beghi E , Helbok R , et al. Global incidence of neurological manifestations among patients hospitalized with COVID‐19 ‐ A report for the GCS‐NeuroCOVID consortium and the ENERGY consortium. JAMA Netw Open. 2021;4 (5):1‐14. 10.2139/ssrn.3759712 PMC8114143 33974053

[27] Rahman A , Niloofa R , Jayarajah U , De Mel S , Abeysuriya V , Seneviratne SL . Hematological abnormalities in covid‐19: a narrative review. Am J Trop Med Hyg. 2021;104(4):1188‐1201. 10.4269/ajtmh.20-1536 33606667 PMC8045618

[28] Terpos E , Ntanasis‐Stathopoulos I , Elalamy I , et al. Hematological findings and complications of covid‐19. Am J Hematol. 2020;95(7):834‐847. 10.1002/ajh.25829 32282949 PMC7262337

[29] Espejo E , Andrés M , Borrallo RM , et al. Bacteremia associated with pressure ulcers: a prospective cohort study. Eur J Clin Microbiol Infect Dis. 2018;37(5):969‐975. 10.1007/s10096-018-3216-8 29479635 PMC5916975

[30] Taylor EH , Marson EJ , Elhadi M , et al. Factors associated with mortality in patients with Covid‐19 admitted to intensive care: a systematic review and meta‐analysis. Anaesthesia. 2021;76(9):1224‐1232. 10.1111/anae.15532 34189735 PMC8444810

