# Corrigendum to “The outcome of COVID‐19 patients in the intensive care unit in Sudan: A cross‐sectional study”

**DOI:** 10.1002/hsr2.1189

**Published:** 2023-04-14

In this article^1^, the title “The outcome of COVID‐19 patients in the intensive care unit in Sudan: A cross‐sectional study” has been corrected to “The outcome of COVID‐19 patients in the intensive care unit in Sudan: A retrospective cohort study.”

We apologize for this error.
